# Vestibular rehabilitation in Europe: a survey of clinical and research practice

**DOI:** 10.1007/s00415-020-10228-4

**Published:** 2020-10-13

**Authors:** Dara Meldrum, Lisa Burrows, Ondrej Cakrt, Hassen Kerkeni, Christophe Lopez, Frederik Tjernstrom, Luc Vereeck, Oz Zur, Klaus Jahn

**Affiliations:** 1 Academic Unit of Neurology, School of Medicine, Trinity College Dublin, Dublin, Ireland; 2 Southport and Ormskirk Ear Nose and Throat Balance Clinic Service, Southport, UK; 3 Department of Rehabilitation and Sports Medicine, Second Faculty of Medicine, Charles University and Motol University Hospital, Prague, Czech Republic; 4 Department of Neurology, Inselspital, Bern University Hospital, University of Bern, Bern, Switzerland; 5 Aix-Marseille University, CNRS, LNSC, Marseille, France; 6 Department of Otorhinolaryngology, Head and Neck Surgery, Lund University, Lund, Sweden; 7 Department of Rehabilitation Sciences and Physiotherapy/Movant, Faculty of Medicine and Health Sciences, University of Antwerp, Antwerpen, Belgium; 8 Ben-Gurion University of the Negev, Beersheba, Israel; 9 Department of Neurology and German Center for Vertigo and Balance Disorders, Schoen Clinic Bad Aibling and LMU Munich, Kolbermoorer Strasse 72, 83043 Bad Aibling, Germany

**Keywords:** Vertigo, Dizziness, Benign paroxysmal positional vertigo, Vestibular assessment, Vestibular education, Vestibular rehabilitation

## Abstract

Vestibular rehabilitation (VR) is practiced across Europe but little in this area has been quantified. The aim of this study was to investigate current VR assessment, treatment, education, and research practices. This was an online, cross-sectional survey with 39 VR specific questions and four sections: demographics, current practice, education, and research. The survey was disseminated through the Dizzynet network to individual therapists through country-specific VR special interest groups. Results were analysed descriptively. A thematic approach was taken to analyse open questions. A total of 471 individuals (median age 41, range 23 − 68 years, 73.4% women), predominately physiotherapists (89.4%) from 20 European countries responded to the survey. They had worked for a median of 4 years (range < 1 − 35) in VR. The majority (58.7%) worked in hospital in-patient or out-patient settings and 21.4% in dedicated VR services. Most respondents specialized in neurology, care of the elderly (geriatrics), or otorhinolaryngology. VR was reported as hard/very hard to access by 48%, with the main barriers to access identified as lack of knowledge of health care professionals (particularly family physicians), lack of trained therapists, and lack of local services. Most respondents reported to know and treat benign paroxysmal positional vertigo (BPPV 87.5%), unilateral vestibular hypofunction (75.6%), and cervicogenic dizziness (63%). The use of vestibular assessment equipment varied widely. Over 70% used high-density foam and objective gait speed testing. Over 50% used dynamic visual acuity equipment. Infrared systems, Frenzel lenses, and dynamic posturography were not commonly employed (< 20%). The most frequently used physical outcome measures were the Clinical Test of the Sensory Interaction of Balance, Functional Gait Assessment/Dynamic Gait Index, and Romberg/Tandem Romberg. The Dizziness Handicap Inventory, Visual Analogue Scale, Falls Efficacy Scale, and the Vertigo Symptom Scale were the most commonly used patient reported outcome measures. Adaptation, balance, and habituation exercises were most frequently used (> 80%), with virtual reality used by 15.6%. Over 70% reported knowledge/use of Semont, Epley and Barbeque-Roll manoeuvres for the treatment of BPPV. Most education regarding VR was obtained at post-registration level (89.5%) with only 19% reporting pre-registration education. There was strong (78%) agreement that therapists should have professionally accredited postgraduate certification in VR, with blended learning the most popular mode. Three major research questions were identified for priority: management of specific conditions, effectiveness of VR, and mechanisms/factors influencing vestibular compensation and VR. In summary, the survey quantified current clinical practice in VR across Europe. Knowledge and treatment of common vestibular diseases was high, but use of published subjective and objective outcome measures as well as vestibular assessment varied widely. The results stress the need of improving both training of therapists and standards of care. A European approach, taking advantage of best practices in some countries, seems a reasonable approach.

**Electronic supplementary material:**

The online version of this article (10.1007/s00415-020-10228-4) contains supplementary material, which is available to authorized users.

## Introduction

Vestibular rehabilitation (VR) is a specialised form of rehabilitation for individuals with physical and psychological impairments due to vestibular disease or dysfunction. The need for VR is evident as vestibular problems are highly prevalent and costly [1–4]. The evidence base for VR has expanded considerably since its origins in the 1940s [5]. Several systematic reviews have concluded efficacy in sub-populations of vestibular dysfunction including unilateral and bilateral peripheral dysfunction [6, 7], post-acoustic neuroma resection [8], middle-age to older adults [9], and post-concussion [10]. Additionally, clinical guidelines developed for the treatment of peripheral dysfunction provided strong recommendations that VR should be offered to patients with unilateral or bilateral peripheral vestibular dysfunction with a preponderance of benefit over harm while acknowledging that not all patients benefit [11]. Further, there is abundant evidence for the efficacy of specific manoeuvres to treat benign paroxysmal positional vertigo (BPPV) since the 1980s [12, 13].

VR is usually provided by therapists [14] (physiotherapists, occupational therapists, and audiologists) but also by physicians [15] and is considered a speciality within these professions. This is appropriate as there are often multiple causes for symptoms of dizziness and imbalance, each of which require accurate diagnosis and separate treatments [16]. Although serious causes of chronic dizziness and vertigo are rare, early identification in acute cases can be life-saving [17]. Furthermore, there are complexities in recovery [18, 19], and many direct and indirect psychological and functional factors to consider [20, 21]. Therefore, knowledge and skill in differentiating presenting complaints and selecting the most appropriate physical assessment and appropriate exercise prescription treatment are of paramount importance.

Previous studies investigating the practice of VR have found low levels of usage by physicians due to lack of access and knowledge [15, 22]. In addition, low levels of pre-registration training in VR across professionals ranging from 4 to 53% have been reported [14, 23, 24]. Surveys of therapists practicing VR are outdated [14], have focussed on just one aspect of VR (BPPV) [23], or geographically confined to one area with a very small sample size [24]. Up to date knowledge about current practice of VR in Europe is scant. It is not known what comprises VR across Europe or whether commonalities exist in approaches to assessment and management. There is little information on training and education as well as research capacity amongst those doing VR. There are also gaps in knowledge about how VR is accessed and how accessible it is. Updating the knowledge base in relation to the practice of VR would be of use to those researching the area and seeking to increase capacity. The aim of this study, therefore, was to generate new knowledge on the practice of VR across Europe.

## Methods

The study was a cross-sectional survey administered online. To be included, respondents had to be practicing as therapists in the area of VR. Those not working in Europe at the time of filling out the survey were excluded, as were those who could not fill it out in either the German or English language.

The study was approved by the School of Medicine’s Research Ethics Committee at Trinity College Dublin, Ireland. The survey link was initially distributed by a gatekeeper based at Trinity College Dublin, to the investigators, all experts in VR, who subsequently forwarded it to their network and to country-specific VR special interest groups (where they existed). The invitation email was also disseminated by the Dizzynet network. The survey was promoted verbally at the annual Dizzynet meeting in October 2019 and also at the 2019 UK Annual Conference on VR (ACPIVR). The survey opened on 13/5/2019 and was closed on 13/11/2019. Consent to participate in the survey was implied by completion and online submission of the survey. In the email, potential participants were explicitly informed that their participation was voluntary and anonymous. An email reminder after 1 week of the initial invitation was sent. A target sample of 120 was planned.

### Survey instrument

The aims and objectives of the survey, and initial content were developed during a focus group meeting at the Dizzynet meeting in Starnberg near Munich in October 2018 (with 7 of the authors in attendance) [25]. Dizzynet is a European network initiative for vertigo and balance research (www.klinikum.uni-muenchen.de/European-Dizzynet). Subsequently, the survey instrument was designed using Google Surveys. It was initially piloted by the authors, further refined and once consensus was reached on content and format, it was sent to ten therapists from eight countries for feedback. It was also translated to German. The survey instrument (Appendix 1) was available in the English and German languages and had 39 questions pertaining to four sections. The majority of questions were closed questions requiring either quantitative data (e.g., number of years working in VR, percentage of time working in VR), dichotomous (e.g., yes/no) or Likert response (e.g., indicating frequency of use of various treatment approaches: never, rarely, sometimes, frequently, very frequently). Three open questions were specifically designed to elicit qualitative information and had unlimited free text boxes for responses. These were first, the question “What are the main barriers to access to VR?” (Section 1), second “What research questions do you think should be prioritised in VR?” (respondents were asked to rank what they perceived to be the top five research questions in order of importance), and third, “Note any issues you think are important for the future of VR”. The survey took approximately 20 min to complete.

Section 1: Demographics. This section had eight questions on age, sex, country of work, number of years qualified, highest level of professional qualification and job grade, present work environment and specialty.

Section 2: Current VR practice. This section was the largest, containing 20 questions. Respondents were asked to self-rate their competency in VR (as either novice, competent, or expert) and to indicate the mode(s) by which patients accessed care with them (family doctor referral, self-referral, consultant physician referral or other health care professional referral). They were asked to quantify the number of years working in VR, the percentage of their current work that was VR, the number of VR patients they managed per week, and the amount of time for initial and follow-up assessments. They were provided with a list of 18 diagnoses and asked to select their knowledge and treatment of these. They were also asked about availability and use of vestibular assessment equipment (such as infrared goggles, posturography, foam mats, video head impulse testing, etc.) and whether they had access to results of vestibular function testing when treating patients. This section also elicited information on most frequently used patient reported and physical outcome measures, the most common modes of treatment (individual, group, phone consultation or telerehabilitation). With regard to treatment, they were asked about knowledge and use of canal repositioning procedures (CRPs) and finally were asked about the frequency of use of different treatment methods (e.g., adaptation, substitution, and habituation exercises).

Section 3: Education in VR. This section had six questions relating to respondent’s undergraduate and postgraduate VR education, the format it took (e.g., peer-to-peer, basic or advanced courses, University accredited courses, etc.) and the levels of work support provided. Respondents were asked to indicate their level of agreement with the statement that therapists should have professionally accredited post-graduate certification for practicing VR. Their opinions on the optimal method of education in this regard were also sought.

Section 4: Research in VR. The final section had five questions. Individual research activity, capacity and interest in involvement in future research was quantified along with an open question on perceived research priorities in VR.

### Data analysis

All data were downloaded from Google Surveys into an Excel Spreadsheet and where applicable, translated into English. Data were cleaned and Stata12 (StataCorp LLC) was used for analysis. Data were analysed descriptively. Interval data were examined for normality using the Shapiro − Wilk test and means and standard deviations used where variables were normally distributed with medians and inter-quartile ranges (IQR) used otherwise. Chi-squared tests were used to investigate associations in the data. Teams of three were assigned to analyse the qualitative responses. For the question on research priorities, a first exploratory phase [26] was conducted where participant’s answers or parts of answers were copy-pasted into an Excel spreadsheet and categorized into subthemes and themes. In addition, word counts (e.g., ‘vestibular migraine’) were carried out which helped in defining the final subthemes. This was done independently by the assessors. In a second phase, the word count was repeated and consensus was obtained for the final result. A similar approach was taken for the two other qualitative questions on respondents’ perceptions of barriers to access and other issues in VR.

## Results

**Fig. 1:**
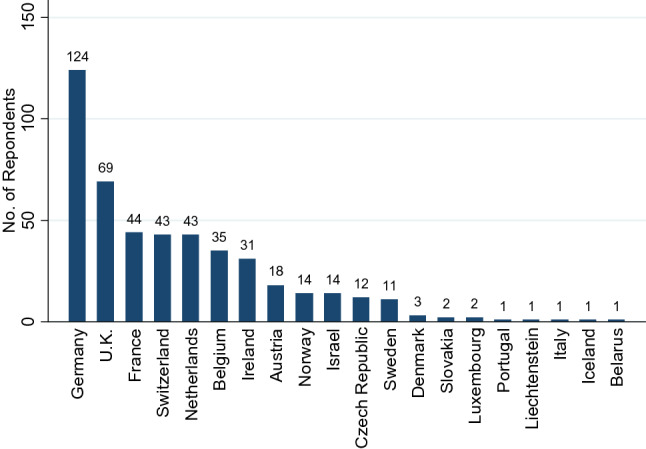
Frequency distribution of countries

A total of 476 responses were received from 22 countries (Fig. 1). The English language version was completed by 64%. Five non-European respondents (*n* = 3 from Australia and *n* = 2 from Argentina) were excluded, but 14 responses from Israel were included. Therefore, a total of 471 surveys from 20 countries were included in the analysis.

### Demographics

The frequency distribution of responses per country is shown in Fig. 1, 50% of countries had 14 or more responses, Germany had the highest number (*n* = 124), followed by the UK (*n* = 69). The median age of respondents was 41 years (range 23–68 years). Demographic data are shown in Table 1. Respondents were mostly female (73.4%) and the predominant profession was physiotherapy (89%). Close to half (50.7%) described themselves as “competent”, 36.3% as “novice”, and 12.9% as “expert” at VR. The median length of time working in VR was 4 years (range < 1–35 years). The majority (62.7%) spent less than 25% of working time in VR with only 11.6% spending greater than 75%. Those who self-reported as “expert” were not qualified longer (i.e., post-registration), but had worked for longer number of years in VR, spent a higher percentage of their working week in VR, and treated more patients per week (Table 2).

**Table 1:** Demographic data *n* = 471

	Median	Min–Max
**Age (years)**	41	23–68
**Gender**	**n**	**%**
Male	125	(26.5)
Female	345	(73.3)
Not given	1	(0.21)
**Highest qualification**	**n**	**%**
BSc	132	28.0
Diploma	95	20.2
MSc	127	27.0
PhD	15	3.2
DPT	8	1.7
Other (not specified)	94	20.0
**Profession**	**n**	**%**
Physiotherapist	421	89.4
Occupational therapist	34	7.2
Orthoptist	5	1.1
Audiological scientist	1	0.2
Chiropractor	3	0.6
Medical doctor	3	0.6
Therapist	2	0.4
Naturopath	1	0.2
RGN	1	0.2
**Job environment**^a^	**n**	**%**
Private practice	252	53.5
Hospital in-patient	140	29.7
Hospital out-patient	134	28.5
Specialist VR service	101	21.4
Rehabilitation centre	59	12.5
Academic institution (research)	40	8.5
Academic institution (teaching)	40	8.5
Residential care	19	4.0
Community/primary care	12	2.5
Other	5	1.1
No answer	2	0.4

*BSc* bachelor of science, *DPT* doctorate in physical therapy, *MSc* master of science, *PhD* doctor of philosophy, *RGN* registered general nurse

^a^Respondents could select > 1

**Table 2:** Level of competency and vestibular rehabilitation professional practice parameters

	All (*n* = 471)		Novice (*n* = 171)		Competent (*n* = 239)		Expert (*n* = 61)	
	Median (IQR)	Min–max	Median (IQR)	Min–max	Median (IQR)	Min–max	Median (IQR)	Min–max
Years post registration	16 (16)	< 1–47	13 (15)	< 1–40	17.5 (15.5)	1–47	17.5 (12)	4–42
Years in VR	4 (4)	< 1–35	1 (1)	< 1–30	5 (7)	< 1–35	14 (11)	2–32
% of working time in VR	15 (25)	0.01–100	10 (10)	< 1–100	20 (30)	1–100	70 (58)	5–100
Number of VR patients per week	4 (8)	0.05–150	2 (2)	0.05–30	5 (8)	1–150	20 (38)	1–150

*VR* vestibular rehabilitation

### Work environments and specialities

**Fig. 2:**
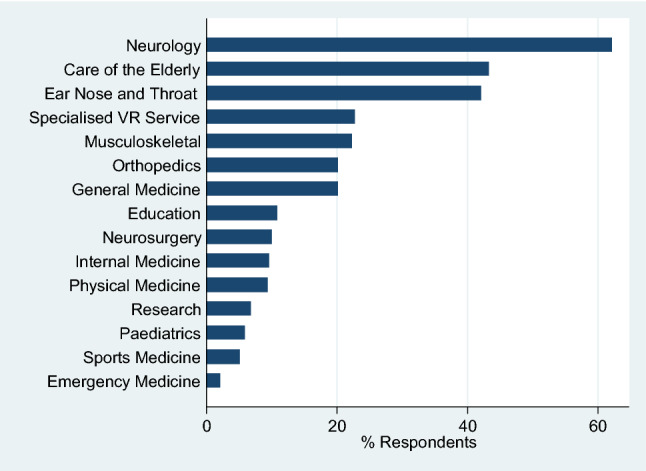
Specialty areas in which vestibular rehabilitation skills were applied

The vast majority of respondents (82.5%) worked in two or less work environments (Table 1). A hospital setting (29.7% in-patient; 28.5% out-patient) predominated but private practice accounted for 53.5%. Over a fifth (21.4%) indicated that they worked at least part of the time in a specialist VR service. The percentage working in private practice varied across countries: 86.3% of French respondents worked in private practice, followed by 60% of Belgians and 57.2% of Germans. Respondents worked in a median of three specialities, most commonly in neurology (62.2%) followed by care of the elderly (43.4) and ENT (42.0%; Fig. 2).

### VR practice

Respondents predominantly received referrals from consultant physicians (67.5%) but also from family doctors (63.1%), other health care professionals (52.4%) and less commonly reported that they accepted patient self-referral (13.5%) as an access route to VR. Regarding consultation times, a median of 40 (IQR, 30) minutes for an initial assessment and 30 (IQR, 5) for a follow-up consultation was reported. A majority (92%) “always” used an individual mode of treatment and telerehabilitation was used by only 4.5%.

Infrequent access to the results of vestibular function assessment was evident, with most reporting they “sometimes” had access (55.5%), and only 27% reporting “always”. The majority (62%) had no vestibular function testing available in their workplace. There was wide variation in access and use of specialised equipment for vestibular assessment (Table 3). Only 40% indicated they “sometimes” or “always” used either Frenzel’s lenses or infrared goggle systems (Table 3). The two most frequently used pieces of equipment were high-density foam and a marked-out distance for gait testing (which were used sometimes or always by > 75%). Computerised posturography (e.g., Balance Master, Equitest, Framiral) was inaccessible to all but 20%. However, 21% sometimes or always used a static force plate. A marked-out Fukuda/Unterberger test (68%), dynamic visual acuity (DVA) testing (58%) and the rotatory chair (40%) were the next most ubiquitously used tests (Table 3).

**Table 3:** Access to and use of vestibular assessment equipment

Equipment	No. responding	Always	Never	No access	Sometimes	Would use if I had access
Infrared goggles with recording	408	10.0	19.6	64.7	4.9	16.2
Infrared goggles, no recording	398	12.1	45.0	37.9	7.0	8.5
Balance: Equitest	394	0.8	21.3	74.4	4.1	12.9
Balance: Balance master	396	1.0	21.2	74.7	5.6	12.1
Static force plate	405	8.9	18.5	62.0	11.9	9.1
Dynamic force plate	396	5.8	19.9	66.2	8.6	12.4
Frenzel lenses	415	9.9	24.8	44.1	20.0	14.2
Subjective visual vertical (bucket test)	398	12.3	23.1	41.2	25.4	6.5
ETDRS	272	7.4	37.5	48.2	8.5	5.1
Snellen	416	18.5	21.4	37.0	26.2	3.6
Computerised gaze stabilisation test	279	5.1	29.7	56.5	6.5	18.8
Video head impulse test	409	10.5	20.5	55.3	19.3	8.8
Treadmill DVA	391	2.3	27.9	60.1	10.2	11.5
Optokinetic drum	405	9.1	24.7	56.3	13.3	7.2
Computerized optokinetic test	275	5.5	29.8	57.1	7.3	14.9
High-density foam	435	45.3	9.0	17.0	31.0	0.7
Computerised gait analysis	393	5.6	24.7	58.8	12.0	11.2
Known and marked out gait test	431	45.2	10.4	13.0	33.4	1.6
Known and marked out Fukuda test	441	37.6	18.1	13.4	30.4	2.3
Rotatory chair	416	15.9	23.6	35.6	25.2	7.2

*DVA* dynamic visual acuity, *ETDRS* early treatment of diabetic retinopathy study

### Conditions treated

**Fig. 3:**
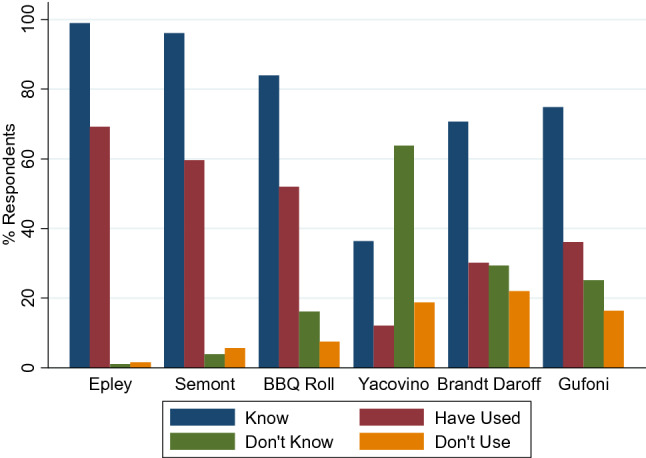
Knowledge and use of canal repositioning procedures

BPPV was reported as known by the vast majority (98.9%) and treated by 87.5% (Table 4). Use of canal repositioning procedures is shown in Fig. 3. The next most commonly treated conditions were unilateral vestibular loss (including vestibular neuritis), dizziness in the elderly (presbystasis), persistent postural-perceptual dizziness (PPPD), and cervicogenic dizziness. The least commonly treated conditions were perilymphatic fistula and vestibular paroxysmia (Table 4).

**Table 4:** Knowledge and treatment of vestibular conditions

Condition	Know%	Treat%	Do not know%	Do not treat%
BPPV	98.9	87.5	1.3	3.0
Vestibular neuritis	93.3	66.2	4.4	14.0
Cervicogenic dizziness	93.3	63.0	4.7	13.6
Unilateral vestibular hypofunction	92.8	75.6	5.1	9.8
Multiple sclerosis	90.0	61.9	6.8	20.1
Traumatic brain injury	89.8	59.2	5.6	21.8
Vestibular migraine	89.2	51.5	7.9	21.7
Functional dizziness	88.9	61.7	9.0	30.6
Presbystasis	85.0	70.2	13.4	16.2
PPPD	84.8	64.7	13.8	19.4
Meniere’s disease	82.7	54.4	1.8	18.0
Post-concussion	78.2	49.5	18.6	26.2
Cerebrovascular accident	76.8	17.5	3.2	16.1
Mal de Debarquement	57.6	27.7	38.5	34.3
Perilymphatic fistula	56.1	14.5	37.2	49.1
Vestibular paroxysmia	52.0	7.3	37.8	40.0

*BPPV* benign paroxysmal positional vertigo, *PPPD* persistent postural-perceptual dizziness

### Types of VR exercises

Beside BPPV manoeuvres, in order of frequency, balance training, adaptation, habituation, and gait retraining exercises were utilised most frequently. Brandt–Daroff exercises, virtual reality, and visual retraining exercises were the least frequently used (Table 5).

**Table 5:** Use of vestibular rehabilitation exercises

Type of exercise	Percentage of respondents				
	Very frequently	Frequently	Sometimes	Rarely	Never
Balance	65.6	26.1	5.7	0.7	2.0
Adaptation	60.5	29.4	6.7	1.7	1.7
Habituation	54.5	29.3	12.1	2.0	2.0
Gait retraining	47.0	27.9	14.3	3.1	7.7
Substitution	40.2	28.5	17.2	6.9	7.2
Muscle strength	18.0	24.1	34.7	12.2	10.9
Optokinetic stimulation	15.2	23.2	26.0	13.8	21.8
Breathing/relaxation	14.4	28.2	31.6	15.5	10.3
Brandt–Daroff	7.3	12.6	25.5	24.5	30.1
Virtual reality	5.5	10.0	21.1	11.4	51.9
Visual (pencil push ups)	5.5	15.9	17.6	16.6	43.9

### Physical and patient-reported outcome measures

A wide variation and total of 48 published physical outcome measures appeared in the top three listed by respondents. The top three reported as most frequently used were Romberg (or Tandem Romberg), the Clinical Test of Sensory Interactions on Balance (CTSIB or mCTSIB), and gait analysis in the form of Dynamic Gait Index (DGI) or Functional Gait Assessment (FGA). The single leg stance test or variant was the next most frequently used followed by the Berg Balance Scale, the Dix–Hallpike test, dynamic visual acuity (DVA) test and Fukuda/Unterberger. Computerised posturography was not used frequently. A detailed list of the physical outcome measures is shown in the supplementary material.

Concerning patient-reported outcome measures (PROMs), respondents were first asked to list the top three PROMs they used and thereafter were provided a list of published PROMs and asked to indicate their usage on a Likert Scale. The most frequently used PROMs cited by respondents were the Dizziness Handicap Inventory (DHI), Vertigo/Dizziness Visual Analogue Scales (VAS) and the Activities Balance Confidence Scale (ABC). An excess of 30 published PROMs were used (see supplementary material). Only two PROMs from the provided list were used “always” or “sometimes” by greater than 50% of respondents. These were the DHI and Dizziness/Vertigo VAS. The next most commonly used PROMs were the ABC, Vertigo Symptom Scale and Falls Efficacy Scale, but were used by less than 30%.

### Access to VR

VR was ranked as “hard” or “very hard” to access by 48% of respondents, “accessible” by 44% and easy to access by only 8%. Israel, Belgium, The Netherlands, and France were the countries with greatest perceived accessibility (> 65% ranked VR as “accessible” or “easy to access”).

### Education in VR

A low percentage (19%) reported that they had pre-registration training in VR, but the large majority (90%) had completed post-registration VR training, with a median of two formats attended and the most common formats being basic and advanced courses in VR. There was broad agreement by 75% with the statement that “therapists should have professionally accredited post-graduate certification for practicing in the area of VR”. A blended learning approach combining web based, clinical and attendance at college/university was most commonly selected as the optimal mode of delivery (56%), followed by clinical training only (25%). Only half of respondents indicated their course tuition was paid and time off from work was supported.

### Research activity and priorities

Just over a fifth of respondents (22.5%) stated they were research active. Of those that were research active, 61% expressed interest in becoming part of a European network on research and 42% indicated capacity to be a trial site. Being research active was significantly associated with increased levels of self-reported competence (*χ*^2^ = 62.1, *p* < 0.0001), interest in becoming part of a European research network (*χ*^2^ = 51.7, *p* < 0.0001) and capacity to be a trial site (*χ*^2^ = 65.4, *p* < 0.0001).

289 therapists out of 471 (61.3%) answered the open question related to research questions that should be prioritized. Some therapists gave multiple suggestions which resulted in 776 counts. Three major themes/research areas were identified: management of specific conditions (317 counts), effectiveness of VR (246 counts), and mechanisms/factors influencing vestibular compensation and dizziness (206 counts). These themes were divided into subthemes of which some were split up in second-order subthemes. The number of counts per subtheme is shown in Table 6.

**Table 6:** Results from thematic analysis of research priorities

Research priorities themes	Counts	
1. Management of dizziness/vertigo in specific conditions	317	
Chronic dizziness/vertigo	62	
Chronic dizziness/vertigo		10
Persistent Perceptual Postural Dizziness (PPPD)		35
Visual vertigo/Visually Induced Dizziness		8
Mal de Debarquement syndrome (MdDS)		6
Motion sickness		3
Benign Paroxysmal Positional Vertigo (BPPV)	45	
Recurrent BPPV		10
Other		35
Vestibular migraine	42	
Ageing and falls (presbyvestibulopathy, multiple sensory deficit)	35	
Menière’s disease/hydrops	23	
Unilateral vestibular hypofunction (vestibular neuritis)	21	
Cervicogenic dizziness	19	
Central nervous disease (stroke, multiple sclerosis)	18	
Traumatic Brain Injury (TBI, concussion, commotion)	16	
Bilateral vestibular hypofunction	14	
Other (children, orthostatic, vestibular paroxysmia)	14	
2. Evaluating effectiveness of vestibular rehabilitation (VR)	246	
Efficacy of different VR protocols in different diseases	61	
Outcome measures	49	
Vestibular function (caloric testing)		5
Balance		6
Impact of disease (quality of life, work)		5
To monitor compensation/progress		3
Other (best?, subjective or objective?, oculomotor testing)		30
Optimal parameters for VR (frequency/duration/length/dosage)	26	
VR-setting (self/individual/group/telerehabilitation/on line)	24	
Incorporation of rehabilitation technology (virtual reality, OKS)	24	
Efficacy of VR compared to other treatments	16	
Psychological treatment in VR (cognitive behavioral therapy, mindfulness)	15	
Timing of VR	11	
Other (medication, multidisciplinary care, different models of care, holistic)	20	
3. Understanding vestibular compensation and dizziness	206	
Models and mechanisms (psychological factors, sensory reweighting, etiology)	83	
Education of physiotherapist (PT)	26	
Care pathways (and how to improve them)	24	
Developing of diagnostic algorithms	18	
Factors influencing compensation	18	
Epidemiology (prevalence of, frequency of)	11	
Role of PT in diagnostics	10	
Other	16	
4. Other (remuneration, artificial labyrinth, differences between countries…)	7	
5. No answer (blank, do not know)	182	

The first theme concerned the management of specific diagnoses. Since some respondents only mentioned the name of the condition, it was not always clear what they meant to investigate. The most commonly reported conditions were chronic dizziness (62 counts), BPPV (45), vestibular migraine (42), and dizziness in the elderly (35).

The second theme concerned the study of the effectiveness of VR. Respondents not only reported a need to assess the effectiveness of VR in general (16 counts), but were also particularly interested in comparing different methods of VR in different diseases (61 counts). In addition, they were interested in the best way of delivering VR, to optimize exercise adherence and how to incorporate rehabilitation technology: timing of VR (11 counts), VR setting [24], VR parameters [26], and rehabilitation technology [24]. Furthermore, they were looking for the optimal way to measure rehabilitation outcome (49 counts).

The third theme focussed on understanding vestibular compensation and dizziness. A large number of therapists expressed the need to better understand mechanisms, models, and aetiologies (83 counts). In addition, questions were asked with a view to improving both the multidisciplinary care (improving care pathways: 24 counts) and the diagnostic process (role of PT in diagnostics: 10 counts; developing diagnostic algorithms: 18 counts). Finally, therapists indicated the need for specific education as well as to finding the best way to improve VR knowledge (26 counts).

182 respondents did not answer the research-related questions. There was no difference between physiotherapists and other professions, but there were clear geographical differences, with a large percentage of French (91%) and British (88%) therapists answering, while only a minority of German (37%) and Austrian (17%) therapists answered this question. When compared to women, there were also more men who answered.

### Other issues in VR

The final question in the survey invited participants to indicate any other relevant issues in VR. 64% (*n* = 303) of respondents provided a response. The responses aligned into three main themes.

1. Delivery of best practice: Training at all stages of pre- and post-graduate education, mentoring, consistency and identification of skilled VR therapists and recruitment opportunities; research into testing protocols and red flags, effective referral pathways, prevention, apps and internet resources/downloads, when to initiate VR and exercise specificity; guidance on condition and treatment knowledge, outcomes measures, condition-specific VR, benefit of clinical psychology, efficacy of methods of treatment delivery, e.g. low cost vs high tech, medication management and benefits of prevention strategies.
2. Delivery of patient services identified the need to improve awareness for vestibular symptoms and disorders among the public and all medical health care professionals. Better knowledge would reduce over testing and improve first contact diagnosis and treatment, e.g. in BPPV. Respondents also focused on improving team interaction (general practitioners, physiotherapists, occupational therapists, audiologists, and clinical psychologists), involvement in patient care (e.g. in emergency vertigo), and referral pathways (timely access, waiting times).
3. Cost implications for VR provision identified lack of evidence and accessibility of resources related to healthcare cost benefits of providing early VR intervention, the overall economic benefit of VR, and the cost of training.

## Discussion

This survey is the largest to date of VR practice and the first to survey multiple countries simultaneously in Europe. Previously, the largest study conducted internationally (through the Bárány Society) was in 2009 and recruited 109 therapists in 19 countries worldwide. Our final sample of 471, confined to Europe will serve to update knowledge on VR practice. The sample was not random, but purposive, with recruitment taking place via researchers’ individual networks, promotion at specialised VR conferences, and specialist VR interest groups at country level. Therefore, it is likely that the findings reflect current practice. Although we only included those working in VR, just 12.9% self-declared as “experts”. This indicated that VR is becoming more widely practiced. It was also evident that VR was practiced across many specialities. This is encouraging as vestibular disorders are prevalent across many specialities and not confined to ENT from where VR historically emerged. Of particular interest was the number of respondents working in care of the elderly settings (43.3%). Vestibular disorders are very frequent and problematic in older individuals [1]. Presbyvestibulopathy [27], recently defined by the Bárány Society as a chronic vestibular syndrome with mild bilateral vestibular deficits, is likely to be ameliorated with VR. It was encouraging to see that VR is emerging as a treatment within this setting. VR was also commonly delivered in private practice in many countries. The finding that a fifth (22.7%) worked within a specialised VR setting is also an indication of growing capacity in the field. A previous study of physiotherapist’s knowledge of BPPV noted that working in a specialist VR environment conferred an advantage in evidence-based awareness [23].

It was an interesting finding that for 62% of respondents, VR usually accounted for less than 25% of workload and, therefore, VR may be considered as a specialty within specialities. This is probably a pragmatic way for VR to be implemented, except for sites where there are tertiary balance and dizziness clinics. It was not surprising, therefore, that the median number of patients treated per week was four with only 25% of respondents treating ten or more patients per week. This has important implications for competency which is generally associated with experience.

In the majority of countries, VR was reported as hard to access. Only 8% of all respondents reported VR as “easy to access”. The barriers to access were consistently reported; limited awareness and knowledge of VR amongst medical doctors and inadequate numbers of trained therapists. These finding agree with a survey of VR which found only a minority of general practitioners (6.8%) referred patients with dizziness to VR [15]. Respondents also reported that VR services were only available in tertiary centres; therefore, long travel distances and extended waiting times were also commonly reported barriers. The costs of VR and limited equipment were also considered as barriers. As the majority used an individual mode of treatment and a significant amount of time for consultation (median of 40 min) the cost implications are obvious. Very few used tele-rehabilitation (4.5%). With the recent COVID-19 pandemic and a rapid move towards telemedicine across all health care areas, it is likely that this now changes. In addition to these barriers, it was concerning to find that there was limited access to the results of vestibular function tests and low use of basic assessment equipment such as infrared goggles.

There was a high level of knowledge and treatment of the most common vestibular disorders: BPPV (98.9%), vestibular neuritis (93.3%), central vestibular disorders (90%), and functional dizziness (88.9%). Of particular interest was the high percentage indicating knowledge and treatment of cervicogenic dizziness (93 and 63%, respectively). The latter is a problematic and controversial diagnosis [28] and physiotherapists who work in VR are well placed to manage all aspects of cervicogenic dizziness. Only 54.4% indicated they were treating patients with Menière’s disease. This is low and may reflect the knowledge that individuals who compensate well between attacks do not require VR. However, VR is not only about vestibular compensation, it also incorporates physical activity, advice and education, all of which are important in Menière’s disease. Similarly, only 51.5% were treating vestibular migraine, possibly reflecting that there is presently weak evidence that VR is effective in the management of this condition [29–31].

### Treatment techniques and outcome measures

Widespread knowledge and use of repositioning manoeuvres were evident, with Epley and Semont manoeuvres being the most frequently known and used ones. The Barbeque Roll and Gufoni manoeuvres were less well known and not as frequently used. This is in agreement with the findings of a BPPV specific study which found that less than half of physiotherapists working in VR could name the test for horizontal canal BPPV [23]. There is a clear need for training and education in this regard as 5–15% of BPPV cases affect the horizontal canal [32]. An encouraging finding was that although 70.7% reported knowing Brandt–Daroff exercises, only 30.2% reported using them, reflecting evidence that Epley is superior to Brandt–Daroff exercises in the treatment of BPPV [12].

A high level of PROM and physical outcome measure use was evident, but with wide variation. The most frequently used PROMs concurred with a recent systematic review of their use in research in VR [33] but the range of tests indicated that there is no consensus so far. A common set of core outcome measures would be of value for clinical studies and comparisons of VR outcomes across jurisdictions.

The types of interventions reported to be used were remarkably consistent across countries. They also closely followed the evidence base. Balance exercises were almost ubiquitously used, as were adaptation and habituation exercises with 2% or less indicating that they never used them. Substitution exercises were not used as frequently as either adaptation or habituation exercises which is in keeping with clinical guidelines, at least for peripheral vestibular hypofunction [11]. It was of interest that optokinetic stimulation which is considered a form of habituation [11] was reported as never or rarely used by 35.6%. Again, this possibly reflects the lack of treatment techniques available for this form of treatment and the lack of evidence supporting its use. Newer forms of technology such as virtual reality were not in widespread use, although reducing costs of these make them conceivable now. However, there is no standard for the use of such systems. Breathing and relaxation exercises were more infrequently used than other interventions. Controlling anxiety that frequently accompanies dizziness and vertigo might advocate their use but the evidence is weak for their effectiveness.

### Research and education

A clear need for improved education at undergraduate level was found with a surprisingly low level of respondents indicating that they received pre-registration VR training. This has been reported by others [14, 24] and recently, presenting a clear need to lobby those in physiotherapy education to consider if this should be rectified. It was encouraging to see that almost all had engaged in postgraduate training. A high level of agreement that postgraduate certification is a worthy aspiration for the field merits consideration by those in postgraduate education. With regard to research in VR, a wealth of information was generated on what therapists considered to be a priority in the area. We intend to use this information to present a position statement on research in VR in Europe in the near future. There is excellent research capacity amongst the countries (1 in 5 participants were research active) and willingness to participate in multi-centre trials which bodes well for the field.

## Limitations

The reliability and validity or the survey were not assessed, but the contribution of almost 500 individual therapists and physician experts in VR lends face validity to the content. Finally, we have no information on non-responders and the sample was not a random one limiting generalisability.

## Conclusion

The survey has resulted in a comprehensive representation of VR practice, education and research in Europe revealing a growing capacity for the VR and broad utilisation of evidence-based intervention for those practicing VR in Europe. There are, however, significant barriers to access and poor access to basic equipment for assessment and to results of vestibular function testing. Increased knowledge and awareness amongst health care professionals and patients alike are key objectives to improve VR as is increasing interactions between physicians and therapists. There is a clear need to improve pre-registration training and to consider how best to formalise post registration training to ensure consistent, evidence based, and harmonised standards in VR in clinic and research.

## Electronic supplementary material

Below is the link to the electronic supplementary material.

Supplementary file1 (DOCX 23 kb)

## References

[1] Agrawal Y, Pineault KG, Semenov YR (2018). Health-related quality of life and economic burden of vestibular loss in older adults. Laryngoscope Investig Otolaryngol.

[2] Agrawal Y, Carey JP, Della Santina CC, Schubert MC, Minor LB (2009). Disorders of balance and vestibular function in us adults: Data from the national health and nutrition examination survey, 2001–2004. Arch Intern Med.

[3] Agrawal Y, Carey JP, Della Santina CC, Schubert MC, Minor LB (2010). Diabetes, vestibular dysfunction, and falls: analyses from the national health and nutrition examination survey. Otol Neurotol.

[4] Neuhauser HK, von Brevern M, Radtke A, Lezius F, Feldmann M, Ziese T (2005). Epidemiology of vestibular vertigo: a neurotologic survey of the general population. Neurology.

[5] Cawthorne T (1946). Vestibular injuries. Proc R Soc Med.

[6] Porciuncula F, Johnson CC, Glickman LB (2012). The effect of vestibular rehabilitation on adults with bilateral vestibular hypofunction: a systematic review. J Vestib Res.

[7] McDonnell MN, Hillier SL (2015). Vestibular rehabilitation for unilateral peripheral vestibular dysfunction. Cochrane Database Syst Rev.

[8] Passier L, Doherty D, Smith J, McPhail SM (2012). Vestibular rehabilitation following the removal of an acoustic neuroma: a systematic review of randomized trials. Head Neck Oncol.

[9] Ricci NA, Aratani MC, Doná F, Macedo C, Caovilla HH, Ganança FF (2010). A systematic review about the effects of the vestibular rehabilitation in middle-age and older adults. Braz J Phys Ther.

[10] Murray DA, Meldrum D, Lennon O (2017). Can vestibular rehabilitation exercises help patients with concussion? A systematic review of efficacy, prescription and progression patterns. Br J Sports Med.

[11] Hall CD, Herdman SJ, Whitney SL, Cass SP, Clendaniel RA, Fife TD (2016). Vestibular rehabilitation for peripheral vestibular hypofunction: an evidence-based clinical practice guideline. J Neurol Phys Ther.

[12] Hilton MP, Pinder DK (2014) The Epley (canalith repositioning) manoeuvre for benign paroxysmal positional vertigo. Cochrane Database Syst Rev 2014(12), Art. No. CD003162. 10.1002/14651858.CD003162.pub3 10.1002/14651858.CD003162.pub3 PMC11214163 25485940

[13] Bhattacharyya N, Gubbels SP, Schwartz SR, Edlow JA, El-Kashlan H, Fife T (2017). Clinical practice guideline: benign paroxysmal positional vertigo (Update). Otolaryngol Head Neck Surg.

[14] Cohen HS, Gottshall KR, Graziano M, Malmstrom EM, Sharpe MH (2009). International survey of vestibular rehabilitation therapists by the Barany Society Ad Hoc Committee on Vestibular Rehabilitation Therapy. J Vestib Res.

[15] van Vugt VA, Diaz Nerio PM, van der Wouden JC, van der Horst HE, Maarsingh OR (2017). Use of canalith repositioning manoeuvres and vestibular rehabilitation: a GP survey. Scand J Prim Health Care.

[16] Maarsingh OR, Dros J, Schellevis FG, van Weert HC, van der Windt DA, ter Riet G (2010). Causes of persistent dizziness in elderly patients in primary care. Ann Fam Med.

[17] Kattah JC, Talkad AV, Wang DZ, Hsieh Y-H, Newman-Toker DEJS (2009). HINTS to diagnose stroke in the acute vestibular syndrome: three-step bedside oculomotor examination more sensitive than early MRI diffusion-weighted imaging. Stroke.

[18] Cousins S, Kaski D, Cutfield N, Arshad Q, Ahmad H, Gresty MA (2017). Predictors of clinical recovery from vestibular neuritis: a prospective study. Ann Clin Transl Neurol.

[19] Schubert MC, Migliaccio AA (2019). New advances regarding adaptation of the vestibulo-ocular reflex. J Neurophysiol.

[20] Dieterich M, Staab JP (2017). Functional dizziness: from phobic postural vertigo and chronic subjective dizziness to persistent postural-perceptual dizziness. Curr Opin Neurol.

[21] Pothier DD, Shah P, Quilty L, Ozzoude M, Dillon WA, Rutka JA (2018). Association between catastrophizing and dizziness-related disability assessed with the dizziness catastrophizing scale. JAMA Otolaryngol Head Neck Surg.

[22] Jayarajan V, Rajenderkumar D (2003). A survey of dizziness management in general practice. J Laryngol Otol.

[23] Male AJ, Ramdharry GM, Grant R, Davies RA, Beith ID (2019). A survey of current management of Benign Paroxysmal Positional Vertigo (BPPV) by physiotherapists' interested in vestibular rehabilitation in the UK. Physiotherapy.

[24] Bush ML, Dougherty W (2015). Assessment of vestibular rehabilitation therapy training and practice patterns. J Community Health.

[25] Jahn K, Lopez C, Zwergal A, Zur O, Cakrt O, Kellerer S (2019). Vestibular rehabilitation therapy in Europe: chances and challenges. J Neurol.

[26] Maguire M, Delahunt B (2017). Doing a thematic analysis: a practical, step-by-step guide for learning and teaching scholars. All Ireland J High Educ.

[27] Agrawal Y, Van de Berg R, Wuyts F, Walther L, Magnusson M, Oh E (2019). Presbyvestibulopathy: diagnostic criteria Consensus document of the classification committee of the Barany Society. J Vestib Res.

[28] Reiley AS, Vickory FM, Funderburg SE, Cesario RA, Clendaniel RA (2017). How to diagnose cervicogenic dizziness. Arch Physiother.

[29] Sugaya N, Arai M, Goto F (2017). Is the headache in patients with vestibular migraine attenuated by vestibular rehabilitation?. Front Neurol.

[30] Bisdorff AR (2011). Management of vestibular migraine. Therapeutic Adv Neurol Disord.

[31] Alghadir AH, Anwer S (2018). Effects of vestibular rehabilitation in the management of a vestibular migraine: a review. Front Neurol.

[32] Bhattacharyya N, Gubbels SP, Schwartz SR, Edlow JA, El-Kashlan H, Fife T (2017). Clinical practice guideline: benign paroxysmal positional vertigo (update) executive summary. Otolaryngol-Head Neck Surg.

[33] Fong E, Li C, Aslakson R, Agrawal Y (2015). Systematic review of patient-reported outcome measures in clinical vestibular research. Arch Phys Med Rehabil.

