# Supplementary material for: Vestibular rehabilitation in Europe: a survey of clinical and research practice
Source: J Neurol. 2020 Oct 13;267(Suppl 1):24–35. doi: 10.1007/s00415-020-10228-4 (PMC7552585; doi:10.1007/s00415-020-10228-4)
Supplement: Supplementary file 1 — Supplementary file1 (DOCX 23 kb) [file 415_2020_10228_MOESM1_ESM.docx]

| **Published Patient Outcome Measures** | **n** | **Always** | **%** | **Sometimes** | **%** | **Never** | **%** | **Not Translated to my language** | **%** |
| --- | --- | --- | --- | --- | --- | --- | --- | --- | --- |
| Dizziness Handicap Inventory | 427 | 140 | **32.8** | 144 | **33.7** | 135 | **31.6** | 8 | 1.9 |
| Visual Analogue Scale of Dizziness/Vertigo | 404 | 103 | **25.5** | 141 | **34.9** | 154 | **38.1** | 6 | 1.5 |
| Activities Balance Confidence Scale | 404 | 36 | **8.9** | 93 | **23.0** | 260 | **64.4** | 15 | 3.7 |
| Vertigo Symptom Scale | 402 | 27 | **6.7** | 110 | **27.4** | 250 | **62.2** | 15 | 3.7 |
| Falls Efficacy Scale | 401 | 24 | **6.0** | 117 | **29.2** | 251 | **62.6** | 9 | 2.2 |
| Vestibular Activities of Daily Living | 399 | 23 | **5.8** | 65 | **16.3** | 291 | **72.9** | 20 | 5.0 |
| Vestibular Rehabilitation Benefits Questionnaire | 402 | 20 | **5.0** | 49 | **12.2** | 306 | **76.1** | 27 | 6.7 |
| Situational Characteristics Questionnaire | 399 | 19 | **4.8** | 47 | **11.8** | 308 | **77.2** | 25 | 6.3 |
| Visual Analogue Scale of Oscillopsia | 391 | 17 | **4.4** | 60 | **15.4** | 304 | **77.8** | 10 | 2.6 |
| Vestibular Ocular Motor Screening | 392 | 15 | **3.8** | 55 | **14.0** | 304 | **77.6** | 18 | 4.6 |
| Hospital Anxiety and Depression Scale | 397 | 13 | **3.3** | 118 | **29.7** | 256 | **64.5** | 10 | 2.5 |
| Disability Rating Scale | 398 | 12 | **3.0** | 49 | **12.3** | 319 | **80.2** | 18 | 4.5 |
| Motion Sensitivity Questionnaire | 398 | 9 | **2.3** | 74 | **18.6** | 295 | **74.1** | 20 | 5.0 |
| Motion Sickness Questionnaire | 397 | 6 | **1.5** | 36 | **9.1** | 333 | **83.9** | 22 | 5.5 |
| UCLA Dizziness Questionnaire | 386 | 4 | **1.0** | 19 | **4.9** | 339 | **87.8** | 24 | 6.2 |
| Beck Depression Inventory | 391 | 1 | **0.3** | 36 | **9.2** | 340 | **87.0** | 14 | 3.6 |

| Number | Physical Outcome Measure | n Reporting Use | % Reporting Use |
| --- | --- | --- | --- |
| 1 | Romberg | 69 | 28.9 |
| 2 | CTSIB | 67 | 28.0 |
| 3 | BBS | 38 | 15.9 |
| 4 | FGA | 35 | 14.6 |
| 5 | DIX HALLPIKE | 33 | 13.8 |
| 6 | DVA | 33 | 13.8 |
| 7 | DGI | 32 | 13.4 |
| 8 | Fukuda/Unterbergers | 29 | 12.1 |
| 9 | TUAG | 28 | 11.7 |
| 10 | BESTTEST/MINIBESTEST | 27 | 11.3 |
| 11 | HIT/vHIT | 22 | 9.2 |
| 12 | VNG | 19 | 7.9 |
| 13 | Gait Analysis (measure not specified ) | 16 | 6.7 |
| 14 | Compurised Posturography/Stabilometry | 15 | 6.3 |
| 15 | 10M WALK | 14 | 5.9 |
| 16 | Oculomotor Testing (measure not specified) | 14 | 5.9 |
| 17 | Tinetti | 10 | 4.2 |
| 18 | SLS | 8 | 3.3 |
| 19 | HSAN | 7 | 2.9 |
| 20 | Rotary chair | 6 | 2.5 |
| 21 | 180/360 degree turn | 5 | 2.1 |
| 22 | 5TSTS | 5 | 2.1 |
| 23 | 6MWT | 2 | 0.8 |
| 24 | BESS test | 2 | 0.8 |
| 25 | Finger Nose Test | 2 | 0.8 |
| 26 | ROM | 2 | 0.8 |
| 27 | TUSS | 2 | 0.8 |
| 28 | VOMS | 2 | 0.8 |
| 29 | audiogram | 1 | 0.4 |
| 30 | CBMS | 1 | 0.4 |
| 31 | Cervical Proprioception | 1 | 0.4 |
| 32 | EMS | 1 | 0.4 |
| 33 | Essen Resources Inventory | 1 | 0.4 |
| 34 | FAC | 1 | 0.4 |
| 35 | FES | 1 | 0.4 |
| 36 | FIM/FAM | 1 | 0.4 |
| 37 | Functional Reach | 1 | 0.4 |
| 38 | Gait Analysis (Marche en Etoile) | 1 | 0.4 |
| 39 | Horizontal Roll Test | 1 | 0.4 |
| 40 | LOS | 1 | 0.4 |
| 41 | MRMI | 1 | 0.4 |
| 42 | NIR | 1 | 0.4 |
| 43 | Rod and Disc Test | 1 | 0.4 |
| 44 | SPPB | 1 | 0.4 |
| 45 | VCOR | 1 | 0.4 |
| 46 | Visual Vertical | 1 | 0.4 |
| 47 | ZBS | 1 | 0.4 |
| 48 | MMSE | 1 | 0.4 |

Modes of Treatment Used by Respondents

| Mode of Treatment | Always Use (%) | Sometimes Use (%) | Never Use (%) |
| --- | --- | --- | --- |
| Individual | 92.0 | 6.9 | 1.1 |
| Group | 3.6 | 24.2 | 72.2 |
| Phone Consultation | 1.5 | 44.0 | 54.5 |
| Telerehabilitation | 0.0 | 5.5 | 94.5 |
